# Differential pathways to disordered eating for immigrant and native adolescents in Taiwan

**DOI:** 10.1186/s40337-023-00781-4

**Published:** 2023-04-03

**Authors:** Duan-Rung Chen, Li-Yin Lin, Brianna Levin

**Affiliations:** 1 Institute of Health Behaviors and Community Sciences, College of Public Health, National Taiwan University, Room 636, No. 17, Xu-Zhou Rd., Taipei, 10055 Taiwan; 2 Department of Leisure Industry and Health Promotion, National Taipei University of Nursing and Health Sciences, 365 MingDe Road, Beitou District, Taipei, 11219 Taiwan; 3 School of Nursing at Johns Hopkins University, 525 N Wolfe St, Baltimore, MD 21205 USA

**Keywords:** Disordered eating, Psychological distress, Weight overestimation, Immigrant adolescents, Weight-teasing

## Abstract

**Background:**

Few studies have investigated disparities in disordered eating between new immigrant and native adolescents in Taiwan. This study examines the differential pathways to disordered eating in these two populations.

**Methods:**

This cross-sectional study analyzed data collected from March to June 2019. In total, 729 adolescents aged between 13 and 16 years recruited from 37 classes in 3 middle schools in New Taipei City were included in the final analysis. Standardized assessment tools measured disordered eating (EAT-26) and psychological distress (BSRS-5). Generalized structural equation modeling was used to conduct the path analysis.

**Results:**

The prevalence of disordered eating was significantly higher in immigrant adolescents than in their native counterparts. Multipath models indicated that weight-teasing driven by overweight and obese status and weight overestimation could lead to disordered eating through psychological distress; however, the pathways differed for the two groups studied. Family weigh-teasing indirectly leads to disordered eating through psychological distress for native adolescents; by contrast, for immigrant adolescents, friend weigh-teasing indirectly leads to disordered eating through psychological distress. Additionally, weight overestimation directly leads to disordered eating and indirectly through psychological distress to disordered eating for immigrant adolescents.

**Conclusion:**

This study offers a plausible explanation of the differences in the paths to disordered eating between immigrant and native adolescents in Taiwan, which was not reported previously. The study urges the need for school-based prevention programs to improve immigrant students’ mental health.

## Abstract (summary)

This study fills the gap in the literature on disparities in disordered eating and factors associated with disordered eating among immigrant adolescents in Taiwan. To our knowledge, this is the first study focusing on disordered eating among immigrant adolescents in Taiwan. The unique contribution of this research is to elucidate the different pathways leading to disordered eating for native and immigrant adolescents. In native adolescents, weight-teasing by family members but not by friends was indirectly related to disordered eating through psychological distress. In adolescents with an immigrant background, weight-teasing by friends was indirectly associated with disordering eating through psychological distress. Additionally, weight overestimation is a unique risk factor directly and indirectly through psychological distress related to disordered eating for immigrant adolescents. As a highly Westernized country, the Taiwanese’s mainstream ideal body shape is “thinness.” Substantial social discrimination against obese adolescents regarding peer acceptance and sexual attraction may put immigrant adolescents at an increased risk of experiencing body dissatisfaction, psychological stress, and eating disorder symptoms due to their minority status, struggling to adapt to a social environment different from their parents, and wanting peer acceptance. The study urges the need for school-based prevention programs to improve immigrant students’ mental health.

## Background

Disordered eating is irregular, including restrained and binge eating [1]. Frequent dieting, compulsive eating behaviors, and chronic weight fluctuations are common characteristics of problematic eating behavior that may progress to eating disorders if left untreated [1]. Disordered eating concerns and behaviors have been documented in various cultures and ethnic groups in the United States and Europe [2–4]. Disordered eating has also increased in several Asian countries [5]. Several studies adopting the EAT-26 revealed that approximately 10.4% and 17.1% of adolescent Taiwanese girls presented with disordered eating behaviors [6, 7]. These prevalence rates are similar to those of teenage girls in South Korea (14.8%) [8] and those of students aged 12–25 years in China [9].

Since 1994, Taiwan’s Southbound Policy concerning China and countries in the Association of Southeast Asian Nations contributed to a substantial increase in transnational marriages in Taiwan [10]. In 2016, the government initiated a new Southbound Policy to promote further international investments and business/trade activities between Taiwan and Southeastern countries, including Vietnam, Thailand, and others [11]. According to the 2019 Ministry of the Interior report, 15.8% of marriages were transnational. Of those transnational marriages, about 74.1% came from Mainland China and Southeast Asian countries, and foreign brides accounted for 72% of transnational marriages [12].

Most new immigrant families are relatively disadvantaged and have lower economic and educational levels. They are often stigmatized as “Problematic minorities” and experience discrimination [13]. Transnational marriage is usually considered unfavorable, a way to escape poverty. Taiwanese men who marry foreign-born women from Southeastern countries often have low socioeconomic status and education levels, most of whom are low-level farmers, workers, or fishermen residing in rural areas [14]. Immigrant mothers usually have high rates of psychological distress (70%) and depression (24%). Their children (sometimes called second-generation immigrants) suffer from physiological or psychological problems [15, 16], compared with children those whose parents are Taiwanese [17, 18]. Immigrant mothers also experience more discrimination than Taiwanese mothers [15, 19].

Acculturation may be defined as social interaction and coping styles that individuals adopt when interacting with individuals and groups from another culture [20]. The acculturation process is often accompanied by psychological stress, known as acculturation stress resulting in adapting from one culture to another [20, 21]. Acculturative tension derives from participating in activities, values, beliefs, and practices of the dominant culture versus the culture of origin [22] and is associated with a host of mental health symptoms such as general psychological distress [23], depression, and suicide [24] and eating disorder symptoms [25]. While several studies indicate the association between the extent of acculturation into the dominant culture and eating disorders in ethnic minority women in the United States [21, 22], few concerns were directed to disordered eating among adolescent immigrants in East Asian countries, including Taiwan. As a highly Westernized society, Taiwan may have an increased risk of body dissatisfaction and disordered eating for adolescents because the ideal body image purported by Western sculpture is much thinner than the actual body size [26]. Minority adolescents who acculturate to Taiwanese culture with a cultural value of thinness may be at a greater risk for experiencing body dissatisfaction and eating disorder symptoms because of their minority status and psychological anxiety from adapting to the dominant culture [27]. However, no research so far has explored this issue. We hypothesize that native and immigrant adolescents might present different pathways to disordered eating because immigrant adolescents may experience additional stress from the acculturation process. Previous studies have indicated that children of immigrant mothers have a higher risk of poor mental health and exhibit more somatic and internalized symptoms than children whose parents are Taiwanese [18, 19].

### Pathways to disordered eating in adolescents

Models of disordered eating have indicated that the pathways to disordered eating are complex and multifaceted [28]. Overweight status and related weight teasing are often the primary factors for disordered eating [29–33]. Numerous studies provided strong evidence indicating peer and family weight-related teasing is associated with disordered eating in adolescents [29–39]; psychological dysfunction resulting from weight-teasing is often considered the pathway leading to disordered eating behaviors [20, 40–47]. For example, a study conducted in China supported the pathway of weight teasing to depression to disordered eating [30]. A Malaysian university student survey also identified that eating disorders could lead to depression through weight teasing [48].

Additionally, the impact of the ideal Western body image on Asian countries is considered a contributing factor to the increasing prevalence of disordered eating in these countries [20, 41]. Bodyweight misperception is a discrepancy between an individual’s actual and self-perceived body weight, including weight underestimation and overestimation [49]. Extensive studies from various cultural contexts, including Taiwan, supports body dissatisfaction as a risk factor for disordered eating [6, 26, 30, 37, 39, 45, 50–52], and body dissatisfaction is closely linked to weight misperception [49, 53, 54]. In particular, evidence supports that weight overestimation, rather than weight underestimation, leads to disordered eating, such as restricted eating [54, 55].

Weight overestimation is related to psychological distress. Studies conducted with American adolescents have reported a significant association between depression and misperception about being overweight or obese [56, 57]. However, longitudinal studies have revealed mixed findings. A survey among American adolescents revealed that depression increased weight overestimation [56]. In contrast, another study conducted among Chinese adolescents indicated that weight overestimation caused an increased risk of depression [53].

### Control variables

Most studies on adolescent eating disorders have involved only women[2, 6, 58–61]. However, recent studies have revealed the existence of eating disorders among men as well [62–64], showing that men have similar prevalence as women in terms of concern about diet, exercise (i.e., exercising compulsively as a means of controlling one’s weight or body shape) and binge eating habits [62–64]. Therefore, sex is controlled in the models.

In addition, as indicated above, most new immigrant families are of lower economic and educational levels [13, 65]. Father’s education is usually used as the family’s socioeconomic index [66], however, The mothers of children from immigrant families were significantly less likely to attain high education than mothers born in Taiwan [28]. Therefore, the models controlled the father’s and mother’s high education. Lastly, the study sample concerns adolescents aged 13–15, and age plays a vital role in everyday life [67]. Therefore, the models also controlled for age.

### The present study

**Fig. 1:**
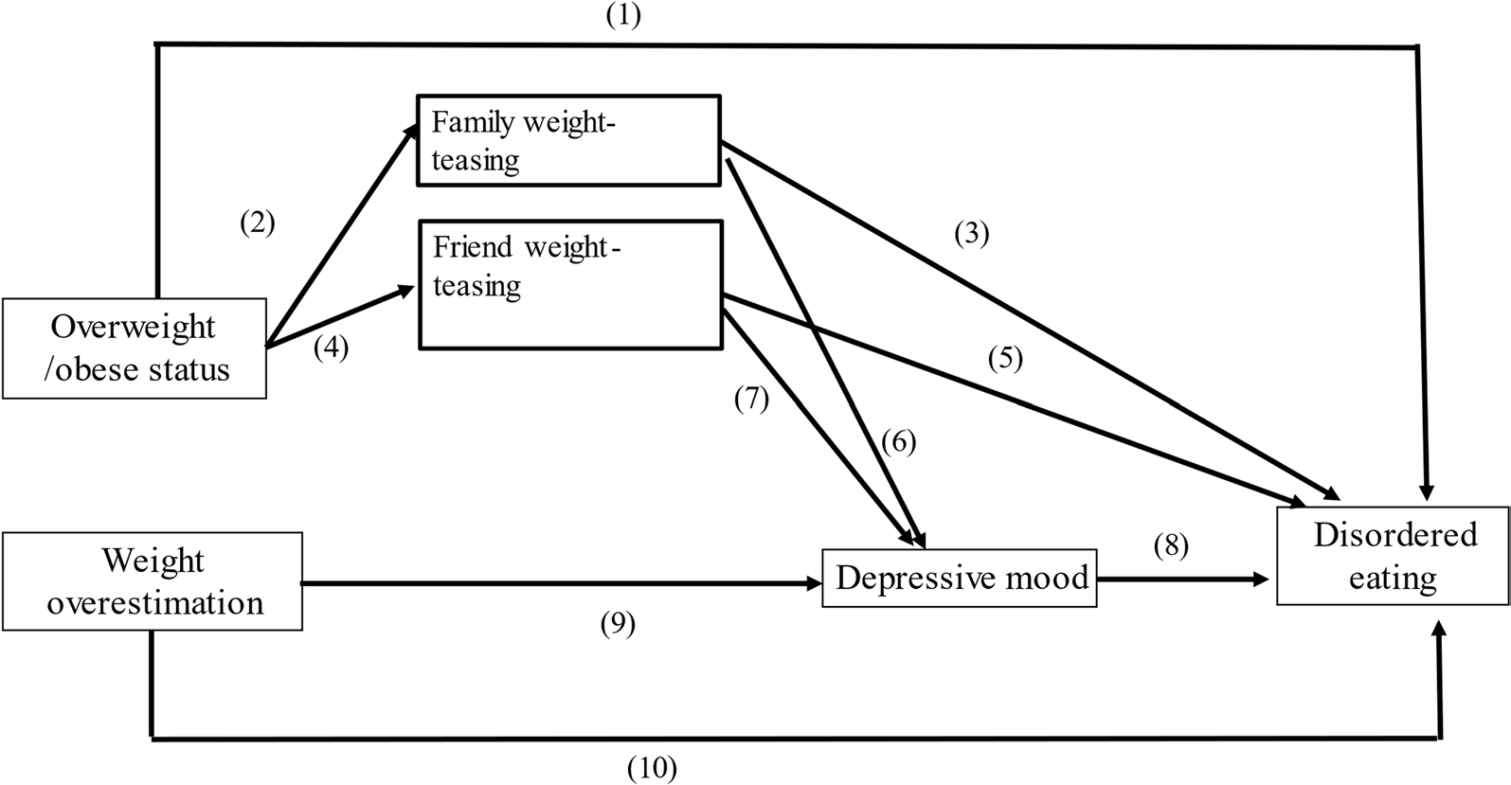
Hypothesized multipath model for disordered eating

Based on previous studies, we hypothesized a multipath model of disordered eating (Fig. 1) and identified ten direct and indirect paths to disordered eating in adolescents, as shown in Fig. 1. However, due to the scarcity of studies on the associations between immigrant adolescents and disordered eating, we first specified the same hypothetical pathways to disordered eating for native and immigrant adolescents. Then, we examined the multipath pathway model for the entire study sample and investigated if being an immigrant adolescent was significantly associated with a high chance of eating disorders. Second, we stratified the study sample into two groups, native and immigrant adolescents, to examine the differences in the pathways to disordered eating for each group.

Path 1 is from overweight/obese status directly to disordered eating. Path 2 is from overweight/obese status to family weight-teasing and disordered eating (path 3). Path 4 is from overweight/obese status to friend weight-teasing and then to disordered eating (path 5). Path 6 is from family weight-teasing to psychological distress, and path 7 is from friend weight-teasing to psychological distress. Path 8 is from psychological distress to disordered eating. Path 9 is from weight overestimation to psychological distress. Path 10 is from weight overestimation directly to disordered eating. The multipath models were controlled for sex, age, and family socioeconomic status (represented by fathers’ education level).

## Methods

### Study participants

This study used a cross-sectional design conducted from March to June 2019 in New Taipei City, Taiwan. The participants were recruited by convenience sampling from 23 classes at three junior high schools in New Taipei City. Three junior high schools were selected through the researchers’ personal contacts, and verbal approvals by the schools’ superintendents and teachers were obtained. Written informed consent forms were collected from the participants and their legal guardians before they participated in this study. Each student completed a 20–25 min questionnaire in a classroom setting.

One hundred fifty-seven participants (15.4%, 157 out of 1020 total participants) were excluded because of incomplete information regarding their parents’ informed consent. Among the 863 valid participants, 134 participants (15.5%) were also excluded from the analysis due to insufficient data on variables of interest. Comparisons between the participants excluded (n = 134) and included (n = 729) in the study indicated no significant difference in demographic characteristics, such as gender, family and friend teasing, family and friend support, and psychological distress state, and EAT-26 scores [29]. A total of 729 participants from 37 classes in 3 middle schools were included for data analysis. The study participants were between 13 and 16 years old, with a mean age of 13.83 years (standard deviation [SD] = 0.98). The study was approved by Taiwan’s Research Ethics Committee (NTU NO. 201901HS030).

### Survey development and measures

A structured self-administered questionnaire was developed for data collection. A prior qualitative study (unpublished work) was conducted in 2018 by interviewing 30 junior high school students in New Taipei City to draft the initial questionnaire. Expert validity was conducted to evaluate the initial questionnaire. Subsequently, the questionnaire was modified based on the suggestions from the three experts. A pretest was then performed among 38 junior high school students to improve the questionnaire further. Finally, the pilot study was conducted among 196 junior high school students to ensure reliability and validity of the final questionnaire [29, 68].

#### Eating attitudes test-26 (EAT-26)

We used the EAT-26 to measure symptoms and characteristics of disordered eating. The EAT-26 comprises 26 questions on disordered eating attitudes and behaviors, and questions are answered using a Likert-type scale, where 0 = never, rarely, or sometimes; 1 = often; 2 = usually; and 3 = always [69]. Disordered eating is indicated by a score of ≥ 20 [69]. Because EAT-26 is an assessment tool widely used to screen for disordered eating among adolescents from various cultural contexts, it was chosen to measure disordered eating in our study participants [6, 20, 37, 41, 52, 70–73].

*Psychological distress* was measured by the Five-item Brief Symptom Rating Scale (BSRS-5). BSRS-5 is a short assessment tool to screen patients and community members for psychological disorders, especially depression and anxiety [74]. The BSRS-5 has been widely used in research on mental health among Chinese and Southeast Asian immigrants in Taiwan [75] and to assess psychopathological symptoms in adolescents [76, 77]. Therefore, we adopted the BSRS-5 to assess adolescents’ psychological distress in our study. The internal consistency of BSRS-5 in this study was satisfactory (Cronbach’s alpha = 0.89). The BSRS-5 contains items measuring “difficulty sleeping,” “feeling nervous,” “feeling easily distressed or angry,” “feeling depressed or in a low mood,” and “feeling inferior.” The BSRS-5 uses a Likert-type scale, where 0 = not at all, 1 = mild, 2 = moderate, 3 = severe, and 4 = extremely severe. Scores ≥ 10 indicate moderate to severe psychological distress.

*Friend weight-teasing* was measured by asking participants whether their friends had made fun of or mocked them about their weight or body shape. Respondents replied with a *Yes or No*.

*Family weight-teasing* was measured by asking participants whether their family members made fun of or mocked them about their weight or body shape. Respondents replied with a Yes or No.

*Weight overestimation* is measured by the difference between respondents’ self-reported weight and height (for calculating BMI) and their perceived body size. Perceived body size was measured by asking respondents, “what do you think of your body size” and respondents replied as underweight, normal, overweight, or obese. BMI was categorized into underweight, normal, overweight, and obese using official sex and gender-specific cutoffs of BMI from Taiwan Health Promotion, Ministry of Health and Welfare [78]. Respondents whose BMI-based body size is normal or underweighted but perceived themselves as overweight and obese were classified as having weight overestimation.

#### Controls

*Immigrant status* Respondents with one or two of their parents who are foreign were classified as immigrant adolescents. This survey question did not specifically ask whether the mother or the father has a foreign nationality. The immigrant adolescents in this study were assumed to be the second-generation immigrant adolescents.

#### Father with high education

Respondents who reported their father’s education as college or above was coded as having a father with high education.

#### Mother with high education

Respondents who reported their mother’s education as college or above was coded as having a mother with high education.

*Age* was considered a continuous variable.

*Sex* referred to the biological distinction between male and female, and coded as 1 for male, 0 for female.

### Statistical analysis

We conducted the univariate analysis to describe the frequencies and means of basic demographic information and factors associated with disordered eating. Moreover, we measured the prevalence rates of disordered eating in all participants and stratified these rates by immigrant status. A chi-square test was used to compare factors associated with disordered eating, and individual items from the EAT-26 were compared between the immigrant and native adolescents. Because the dependent and mediating variables are categorical, we conducted a path analysis using generalized structural equation modeling (GSEM) to test our hypotheses. Indirect and total effects were unavailable because the dependent variable is categorical [79]. The Goodness fit index was also not reported in GSEM models. Therefore, we documented 2000 bootstrapping adjusted odds ratios (AOR) and 95% confidence interval for each path [79].

We created a multipath model separately for immigrant and native adolescents. All statistical analyses were performed using Stata. STATA is a general-purpose statistical software package developed by Stata Corp for data manipulation, visualization, statistics, and automated reporting. The current version is Stata 17, released in April 2021.

## Results

### Univariate and bivariate analysis

The mean age of the study participants was 13.83 years (SD ± 0.98), with girls and boys constituting 51.03% (n = 372) and 48.97% (n = 357) of the sample, respectively (Table 2). Of the participants, 24.42% (n = 178) were immigrants, whereas 75.58% (n = 551) were Taiwanese. A disordered eating prevalence rate of 11.39% was observed among the participants, with disordered eating being defined by a score of ≥ 20 on the EAT-26 (mean = 11.68, SD ± 8.38). The prevalence of disordered eating was 16.85% (mean EAT-26 score = 12.56, SD ± 8.85) among immigrant adolescents and 9.62% (mean EAT-26 score = 11.40, SD ± 8.21) among native Taiwanese adolescents. The immigrant adolescents had a significantly higher prevalence of disordered eating (X^2^ = 6.98, *p* = 0.01) and mean EAT-26 score (X^2^ = 70.09, *p* = 0.01) than their Taiwanese counterparts. Approximately 16.32% (n = 119) and 8.02% (n = 58) of our study participants exhibited moderate to severe psychological distress and bodyweight misperceptions, respectively. Furthermore, relative to Taiwanese adolescents, a significantly lower percentage of immigrant adolescents had mothers who received higher education (30.85% vs. 19.66%; X^2^ = 8.33, *p* = 0.00). Details are presented in Table 1.

**Table 1:** Sample descriptives (N = 729)

	All adolescents		Native adolescents		Immigrant adolescents		X^2^ (df)	*p*-value
Variables	N/Means	%/SD	N/Means	%/SD	N/Means	%/SD		
Age	13.83	0.98	13.82	0.98	13.86	1.00	0.20 (3)	0.98
Sex								
Male	357	48.97	271	49.18	86	48.31	0.04 (1)	0.84
Female	372	51.03	280	50.82	92	51.69		
Parents of a foreign nationality								
Yes	178	24.42	–	–	–	–		
No	551	75.58						
Disordered eating (EAT-26)								
Yes	83	11.39	53	9.62	30	16.85	6.98 (1)	0.01
Mean EAT-26 score	11.68	8.38	11.4	8.21	12.56	8.85	70.09 (43)	0.01
Moderate to severepsychological distress (BSRS-5)								
Yes	119	16.32	84	15.25	35	19.66	1.92 (1)	0.17
Body overestimation								
Yes	58	8.02	43	7.88	15	8.47	0.07 (1)	0.80
Friend weight-teasing								
Yes	184	25.24	134	24.32	50	28.09	1.01 (1)	0.31
Family weight-teasing								
Yes	173	23.73	129	23.41	44	24.72	0.13 (1)	0.72
Mother with college degree or above								
Yes	205	28.1	170	30.9	35	19.7	8.33 (1)	0.004
Father with college degree or above								
Yes	229	32.62	172	32.39	57	33.33	0.05 (1)	0.82

As indicated in Table 2, no significant difference was observed in most of the items in the EAT-26 between immigrant and native adolescents. However, a considerably higher percentage of immigrants than native adolescents (18.54% vs. 11.62%) reported being aware of the calories consumed in food (X^2^ = 5.59, *p* = 0.02).

**Table 2:** The eating attitudes test-26 (N = 729)

	All		Immigrant adolescents		Native adolescents		X^2^ (df)	*p*-value
Items	N1	%	N	%	N	%		
1. I am terrified about being overweight	261	35.80	69	38.76	192	34.85	0.90 (1)	0.34
2. I avoid eating when I am hungry	139	19.07	36	20.22	103	18.69	0.20 (1)	0.65
3. I find myself preoccupied with food	121	16.60	28	15.73	93	16.88	0.13 (1)	0.72
4. I have gone on eating binges where I feel that I may not be able to stop	65	8.92	16	8.99	49	8.89	0.00 (1)	0.97
5. I cut my food into small pieces	170	23.32	39	21.91	131	23.77	0.26 (1)	0.61
6. I aware of the calorie content of foods that I eat	97	13.31	33	18.54	64	11.62	5.81 (1)	0.12
7. I particularly avoid food with a high carbohydrate content (i.e. bread, rice, potatoes, etc.)	71	9.74	20	11.24	51	9.26	0.60 (1)	0.44
8. I feel that others would prefer if I ate more	207	28.40	56	31.46	151	27.40	1.09 (1)	0.30
9. I vomit after I have eaten	11	1.51	3	1.69	8	1.45	0.05 (1)	0.82
10. I feel extremely guilty after eating	47	6.45	16	8.99	31	5.63	2.52 (1)	0.11
11. I am occupied with a desire to be thinner	301	41.29	80	44.94	221	40.11	1.30 (1)	0.26
12. I think about burning up calories when I exercise	232	31.82	59	33.15	173	31.40	0.19 (1)	0.66
13. I other people think that I am too thin	210	28.81	49	27.53	161	29.22	0.19 (1)	0.67
14. I am preoccupied with the thought of having fat on my body	267	36.63	73	41.01	194	35.21	1.95 (1)	0.16
15. I take longer than others to eat my meals	126	17.28	31	17.42	95	17.24	0.00 (1)	0.96
16. I avoid foods with sugar in them	101	13.85	26	14.61	75	13.61	0.11 (1)	0.74
17. I eat diet foods	28	3.84	6	3.37	22	3.99	0.14 (1)	0.71
18. I feel that food controls my life	77	10.56	18	10.11	59	10.71	0.05 (1)	0.82
19. I display self-control around food	292	40.05	73	41.01	219	39.75	0.09 (1)	0.77
20. I feel that others pressure me to eat	95	13.03	25	14.04	70	12.70	0.21 (1)	0.64
21. I give too much time and thought to food	80	10.97	19	10.67	61	11.07	0.02 (1)	0.88
22. I feel uncomfortable after eating sweets	54	7.41	19	10.67	35	6.35	3.66 (1)	0.06
23. I engage in dieting behavior	62	8.50	15	8.43	47	8.53	0.00 (1)	0.97
24. I like my stomach to be empty	59	8.09	17	9.55	42	7.62	0.67 (1)	0.41
25. I have the impulse to vomit after meals	31	4.25	8	4.49	23	4.17	0.03 (1)	0.85
26. I enjoy trying new rich foods	549	95.75	133	95.51	416	95.85	0.04 (1)	0.83

N is the number of participants who reported they have this attitude or behaviors often, most of the time, and always to the questions of EAT26

Column percentages were reported

### Generalized path model for all adolescents

**Fig. 2:**
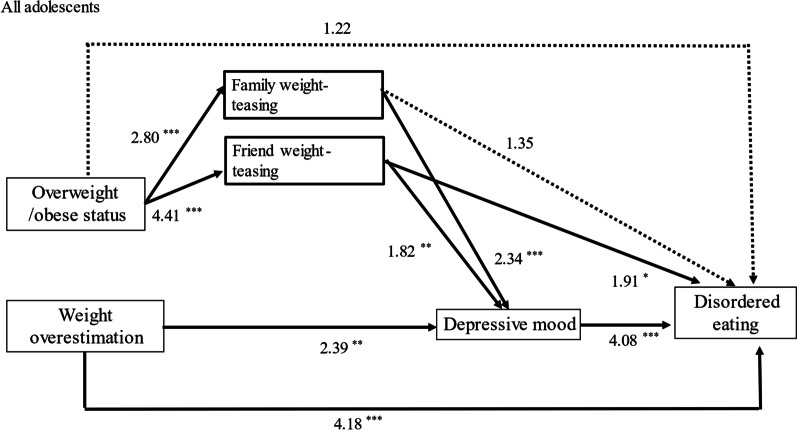
Path analysis results for all adolescents. Adjusted odds ratios (AOR) are presented. *p < 0.05, **p < 0.01, ***p < 0.001. Solid lines represent statistically significant associations. Dotted lines represent statistically insignificant associations

The results revealed that overweight or obese status was significantly associated with negative social interactions, including family teasing and friend teasing (adjusted odds ratio [AOR] = 2.80, *p* < 0.001; AOR = 4.41, *p* < 0.001, respectively). Friend teasing is significantly associated with the likelihood of disordered eating (path 5). Family and friend teasing were significantly related to the likelihood of psychological distress (AOR = 2.34, *p* < 0.001; AOR = 1.91, *p* < 0.001, respectively). Psychological distress was significantly related to the likelihood of disordered eating (AOR = 4.08, *p* < 0.001). As indicated, the pathway from overweight or obesity to disordered eating mainly proceeded through experiencing family and friend teasing, resulting in psychological distress and increasing the likelihood of disordered eating (path 2 → 6 → 8; path 4 → 7 → 8). Another pathway from overweight or obesity led to friend teasing, increasing the likelihood of disordered eating (path 4 → 5). However, the direct pathway from overweight or obesity to disordered eating was not statistically significant (path 1), as shown in Table 3 and Fig. 2.

**Table 3:** Generalized path analysis results

	All adolescents				Native adolescents				Immigrant adolescents			
Pathways	Coef	AOR	95% CI	*p*-value	Coef	AOR	95% CI	*p*-value	Coef	AOR	95% CI	*p*-value
Overweight/Obese status → Disordered eating (1)	0.20	1.22	(0.66, 2.31)	0.54	0.41	1.51	(0.69, 3.31)	0.31	− 0.45	0.64	(0.18, 2.19)	0.47
Overweight/Obese status → Family teasing (2)	1.03	2.80	(1.96, 4.01)	0.00***	1.09	2.97	(1.96, 4.51)	0.00***	0.86	2.36	(1.16, 4.79)	0.02*
Family weight-teasing → Disordered eating (3)	0.30	1.35	(0.74, 2.48)	0.33	0.56	1.76	(0.85, 3.63)	0.13	− 0.24	0.79	(0.24, 2.58)	0.69
Overweight/Obese status → Friend weight-teasing (4)	1.48	4.41	(3.09, 6.31)	0.00***	1.45	4.27	(2.82, 6.49)	0.00***	1.56	4.76	(2.36, 9.62)	0.00***
Friend weight-teasing → Disordered eating (5)	0.64	1.91	(1.03, 3.54)	0.04*	0.49	1.64	(0.76, 3.51)	0.21	1.06	2.88	(0.92, 9.09)	0.07
Family weight teasing → Depressive mood (6)	0.85	2.34	(1.46, 3.76)	0.00***	0.86	2.36	(1.37, 4.08)	0.00***	0.88	2.40	(0.89, 6.48)	0.08
Friend weight-teasing → Depressive mood (7)	0.60	1.82	(1.14, 2.92)	0.01**	0.40	1.50	(0.87, 2.59)	0.15	1.09	2.98	(1.12, 7.92)	0.03*
Depressive mood → Disordered eating (8)	1.41	4.08	(2.30, 7.22)	0.00***	1.24	3.46	(1.75, 6.87)	0.00***	1.74	5.72	(1.77, 18.47)	0.00***
Weight overestimation → Depressive mood (9)	0.87	2.39	(1.27, 4.48)	0.01**	0.68	1.98	(0.92, 4.27)	0.08	1.48	4.40	(1.48, 13.08)	0.01**
Weight overestimation → Disordered eating (10)	1.43	4.18	(1.91, 9.18)	0.00***	0.98	2.67	(0.93, 7.71)	0.07	2.39	10.91	(2.80, 42.53)	0.00***
*Controls*												
Parents of a foreign nationality → Disordered Eating	0.56	1.75	(1.02, 3.02)	0.04*								
Sex (female) → Disordered Eating	− 0.20	0.82	(0.50, 1.34)	0.43	− 0.03	0.97	(0.52, 1.79)	0.91	− 0.71	0.49	(0.19, 1.29)	0.15
Age → Disordered eating	− 0.30	0.74	(0.57, 0.97)	0.03*	− 0.39	0.68	(0.47, 0.97)	0.03*	− 0.21	0.81	(0.50, 1.34)	0.42
Mather's high education → Disordered eating	0.48	1.61	(0.82, 3.15)	0.16	0.63	1.88	(0.86, 4.09)	0.11	0.13	1.14	(0.29, 4.49)	0.85
Father's high education → Disordered eating	0.20	1.23	(0.64, 2.36)	0.54	− 0.04	0.96	(0.43, 2.12)	0.92	0.63	1.88	(0.56, 6.31)	0.31

2000 Bootstrap samples

*p < 0.05, **p < 0.01, ***p < 0.001

Weight overestimation was significantly associated with the likelihood of psychological distress (AOR = 2.39, *p* < 0.01), and psychological distress was associated with an increased chance of disordered eating (AOR = 4.08, *p* < 0.001) (path 9 → 8). Furthermore, weight overestimation was directly associated with an increased likelihood of disordered eating (AOR = 4.18, *p* < 0.0001)(path 10). Finally, being an immigrant adolescent (AOR = 1.75, *p* < 0.04) was significantly associated with an increased likelihood of disordered eating. Details are presented in Table 3 and Fig. 2. We further stratified the study participants by their immigrant status.

### Native adolescents

The results indicate that overweight or obese status was significantly associated with family and friend weight-teasing (AOR = 2.97, *p* < 0.001; AOR = 4.27, *p* < 0.001). However, only family weight teasing was significantly associated with an increased likelihood of psychological distress (AOR = 2.36, *p* < 0.001). Psychological distress was significantly related to an increased chance of disordered eating (AOR = 3.46, *p* < 0.001). Consequently, the pathways from overweight or obese status to disordered eating mainly proceeded from experiencing family weight-teasing, which led to psychological distress, increasing the likelihood of disordered eating (path 2 → 6 → 8). The direct pathway from overweight or obese status to disordered eating was nonsignificant for native adolescents (path 1).

**Fig. 3:**
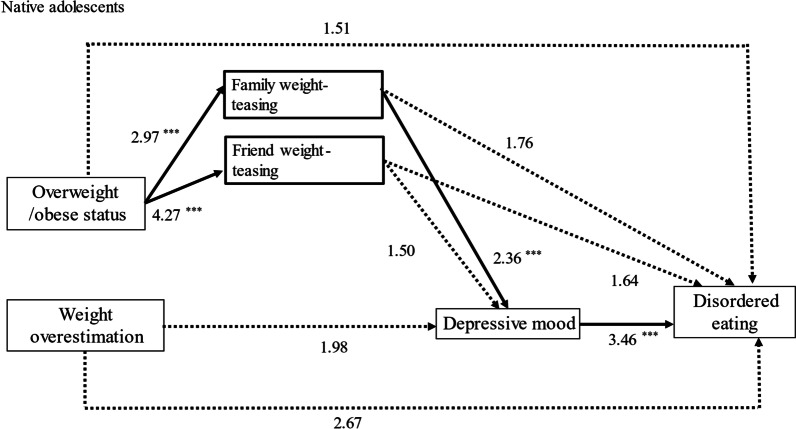
Path analysis results for native adolescents. Adjusted odds ratios (AOR) are presented. *p < 0.05, **p < 0.01, ***p < 0.001. Solid lines represent statistically significant associations. Dotted lines represent statistically insignificant associations

Notably, weight overestimation was unrelated to the likelihood of psychological distress or disordered eating for native adolescents. Details are presented in Table 3 and Fig. 3.

### Immigrant adolescents

Unlike Taiwanese native adolescents, for immigrant adolescents, being overweight or obese was significantly associated with friend weight-teasing (AOR = 4.76, *p* < 0.001). Friend weight-teasing was significantly associated with an increased chance of psychological distress (path 6) (AOR = 2.98, *p* < 0.001). The psychological distress then contributed to an increased likelihood of disordered eating (path 8) (AOR = 5.72, *p* < 0.001). In summary, the pathways from being overweight or obese to disordered eating mainly proceeded indirectly through experiencing friend teasing, not family weight-teasing, which resulted in psychological distress, increasing the likelihood of disordered eating (path 4 → 7 → 8). Like native adolescents, the path from overweight or obese status to disordered eating (path 1) was not statistically significant.

**Fig. 4:**
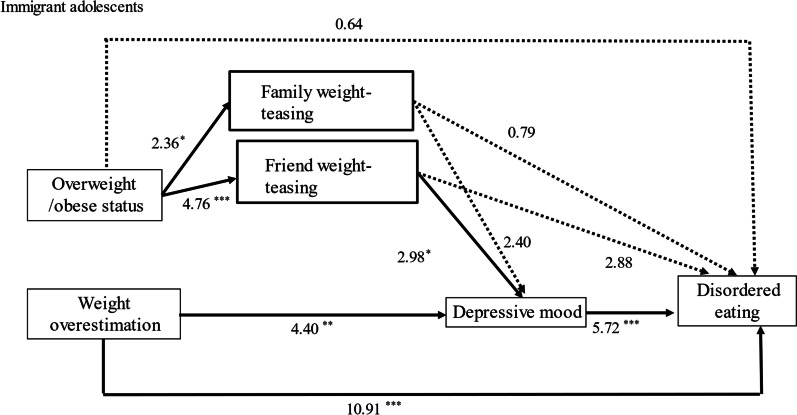
Path analysis results for immigrant adolescents. Adjusted odds ratios (AOR) are presented. *p < 0.05, **p < 0.01, ***p < 0.001. Solid lines represent statistically significant associations. Dotted lines represent statistically insignificant associations

In addition, weight overestimation was directly associated with disordered eating (path 10) (AOR = 10.91, *p* < 0.001) and indirectly proceeded through psychological distress to disordered eating among immigrant adolescents (path 9 → 8). Details are presented in Table 3 and Fig. 4.

## Discussion

This study revealed a significantly higher frequency of disordered eating among immigrant adolescents than their native peers. Studies comparing the prevalence of disordered eating between immigrant and native adolescents in Western countries have reported mixed results on whether immigrant adolescents are at a higher risk for disordered eating [52, 72, 73]. Our study observed that immigrant adolescents in Taiwan have a higher risk of disordered eating compared to their native counterparts.

The unique finding of this research is that, for most adolescents, the path from overweight or obese status to disordered eating is mainly through indirect pathways; however, the pathways are different between these two native and immigrant groups. As for native adolescents, the pathways from overweight or obese led to family teasing, which led to psychological distress, resulting in an increased chance of disordered eating. By contrast, for immigrant adolescents, the indirect pathway was from overweight or obese led to friend weight-teasing, which led to psychological distress, yielding an increased likelihood of disordered eating. Our study helps fill the literature gap regarding the potential risk factors of disordered eating among immigrant adolescents in East Asia.

For native and immigrant adolescents, being overweight or obese is not directly associated with the risk of disordered eating. However, it can significantly increase the risk of weight teasing and, at worst, discrimination [80]. Adolescents who are overweight or obese often experience weight teasing from multiple sources such as schools, healthcare organizations, media, and, most importantly, peers and family members [81]. Independent from weight status, in a school and population-based study, about 28.7% of adolescent girls and 16.1% of teenage boys reported being teased about weight by someone in their families [82].

Our findings were consistent with the vast body of literature in which weight-based teasing has been identified as a critical risk factor for disordered eating among adolescents in both Western and Asian countries [30, 31, 33, 35–37, 39]. In general, there are two types of teasing: weight/shape related teasing vs. overall appearance/non-weight related, and the source of negative comments is often received from parents or peers [83]. The impact of teasing from parents, family members, and peers on body dissatisfaction and disordered eating has been confirmed previously. A study of middle school girls indicated that parental weight-based teasing was a significant predictor of body dissatisfaction and disordered eating behaviors [84]. These negative experiences with body size judging can indirectly increase the likelihood of disordered eating through psychological distress.

Our study discovers a unique perspective where the association between disordered eating and weight teasing differs for native and immigrant adolescents. Our findings found that family teasing can lead to psychological distress, resulting in a high chance of disordered eating for most native Taiwanese adolescents. Similarly, Yu and Perez’s study [39] showed that weight-teasing from mothers was strongly associated with disordered eating in Asian Americans [39]. Chen et al. [68] reported that family members’ weight-teasing was associated with eating disordered among Taiwanese girls. A longitudinal study indicated a significant association between family weight teasing and appearance anxiety [63]. A prospective cohort study conducted on adolescents residing throughout the US found a significant association between parental weight-related teasing and binge eating [85]. These findings suggest the importance of the family environment in developing disordered eating among adolescents. Health promotion programs targeting adolescents’ health should, thus, further examine the family dynamics while designing and implementing family-based interventions for native adolescents.

On the other hand, friend weight teasing was indirectly related to disordered eating through experiencing depression for immigrant adolescents. Friend weight-teasing may result from the social norms regarding ideal body size and exert additional psychological stress on immigrant adolescents while they try to adapt to mainstream society [20, 21]. Further, one study on 80 obese children found that overweight children who experience peer teasing have higher levels of depression and disordered eating than those who are not teased by peers [86]. Acculturative stress is a unique stress often experienced by immigrants navigating and negotiating through different cultures and environments [87]. The stressors resulting from the acculturation process often impair one’s physical, social, and mental health [21, 88]. Furthermore, the cultural body idealization of being thin becomes a critical influencing factor on immigrant adolescents’ body weight perception in most Westernized countries [89]. A literature review on Chinese American women’s experiences with body weight teasing revealed that social acceptance is a crucial factor affecting body weight misperception for Asian immigrants [49]. Studies across cultures found that peer acceptance provides adolescents with better adjustment in school. In contrast, negative peer interactions, specifically peer rejection or isolation, may represent risk factors for poorer psychological functioning, such as depression and maladjustment [90]. Therefore, school-based intervention programs should reduce weight-related teasing and peer criticism.

Notably, no significant association was observed between body weight overestimation, psychological distress, and disordered eating for most native adolescents. However, for immigrant adolescents, weight overestimation significantly increases the risk of disordered eating directly and indirectly through psychological distress. This finding suggests that as a highly Westernized country, Taiwanese’s notion of the ideal body shape is to be “thin” [26, 52]. Evidence revealed that the Taiwanese present substantial social discrimination against obese adolescents in terms of peer acceptance [91], wage differentials [92], and sexual attraction [93]. Many adolescents, in particular those who are overweight or obese, report being teased about their weight and being bothered by the teasing [32]. The perception of being overweight during adolescence is a significant risk factor for depression, and these weight-related discriminatory experience among adolescents is often associated with disordered eating behaviors [80].

Considering being the minority, the parents of immigrant adolescents who moved to Taiwan, where being thin is mainstream, may be at an increased risk for experiencing body dissatisfaction, psychological stress, and eating disorder symptoms due to their minority status and wanting peer acceptance [27]. Racial discrimination against immigrant adolescents in Taiwan may contribute to our study’s main finding. A survey conducted among Southeast Asian and Chinese immigrant women living in Taiwan revealed that racial discrimination was associated with depression [15]. Poor socioeconomic status and the stress related to immigration and acculturation often negatively impact adolescents’ mental health [94]. However, limited research has been conducted on the effect of immigration and acculturation on stress-externalizing behaviors, such as disordered eating. Additionally, in our sample of predominantly Taiwanese individuals of Han Chinese descent, most immigrant families consisted of a mother from China, Vietnam, or Indonesia married to a Taiwanese man of Han Chinese ethnicity. In our data, the disparity between the educational levels of fathers and mothers in immigrant families was significant (*p* < 0.005). Whether the high prevalence of disordered eating is related to stress from the immigrant family environment or simply during the acculturation process is an interesting question for further investigation.

There appears to be a notable association between acculturation and disordered eating psychopathology among immigrant adolescents. Adolescence is a unique period in one’s lifetime where an individual transitions from puberty to adulthood, characterized by marked psychological changes, development of sexual attraction, and efforts toward constructing identity [95]. At the same time, immigrant adolescents in this study struggling to adapt to a social environment different from their parents may be at a higher risk of developing depressive symptoms [96]. Furthermore, the relationship between depression and eating disorders is rather complex because depression can cause a person to struggle with unhealthy eating patterns. In contrast, eating disorders can also lead to feelings of depression [97]. Consequently, these findings emphasize the urgent need for school-based prevention programs to improve immigrant students’ mental health.

This is the first quantitative study assessing the complex relationships between overweight or obese status, weight-based teasing, weight misperception, and disordered eating among immigrant and native adolescents. Public health practitioners should consider the unique characteristics of native and immigrant adolescents when designing specific prevention strategies for disordered eating behaviors for these two populations. Specifically, school-based prevention programs for immigrant adolescents should address body size misconceptions, racial discrimination, and depression associated with weight-based teasing at school. Future research is needed to understand immigration-related risk and protective factors at individual and school levels. As for native adolescents, disordered eating prevention campaigns may focus on understating family dynamics and educating parents on child health rather than weight to reduce peer weight teasing and its harmful consequences, such as disordered eating, depression, substance use, and stress. In addition, we suggest that healthcare providers working with families be aware that parental weight concerns might contribute to family weight teasing and try to use sensitive and non-stigmatizing language when communicating about weight with adolescents and their families.

Our study has several strengths. This study fills the gap in the literature on disparities in disordered eating and factors associated with disordered eating among immigrant adolescents in Taiwan. To our knowledge, this is the first study focusing on disordered eating among immigrant adolescents in Taiwan. We used standardized measures for disordered eating and psychological distress, which allows for international comparisons regarding the potential risks associated with adolescent disordered eating. Lastly, our study offers a plausible explanation of the differences in the paths to disordered eating between immigrant and native adolescents in Taiwan, which was not reported previously.

Some limitations need to be mentioned. First, cross-sectional research cannot be used to infer causality; thus, further research on this topic in Taiwan should be conducted using a study design suitable for determining the basis. Second, we applied convenience sampling in this study, and only adolescents in New Taipei City were recruited. Furthermore, the percentage of immigrant adolescents in the study sample (24.4%) is higher than that of the entire New Taipei City (2017–2019, 11%) [98]. Therefore, the results cannot be generalized to all immigrant and native adolescents in New Taipei City or Taiwan. Future research should investigate social and individual factors associated with disordered eating in these two populations in Taiwan. Lastly, detailed immigration information, including the country where respondent’s parents immigrated from and whether their grandparents also immigrated to Taiwan, is unclear. It may limit our understanding of the family dynamics related to the cause of disordered eating. To expand on the present quantitative results, qualitative studies on disordered eating among immigrant adolescents can be suggested to examine other factors associated with disordered eating within this unique population.

## Conclusion

This study reveals a higher frequency of disordered eating and a higher mean EAT-26 score among immigrant adolescents compared to native adolescents in Taiwan. The proposed pathways to disordered eating appear different for immigrant and native adolescents. Our findings highlight the importance of further research to investigate multi-level factors, such as family, school, and societal factors, associated with disordered eating among immigrant adolescents to prevent disordered eating in this high-risk population. Since health inequality may exist in this case, healthcare providers should carefully consider the differential factors for native and immigrant adolescents separately when designing and implementing disordered eating prevention programs in Taiwan.

## Data Availability

Data is available upon reasonable request.

## Abbreviations

EAT-26: The eating attitudes test-26
BSRS-5: Five-item brief symptom rating scale
AOR: Adjusted odds ratio
GSEM: Generalized structural equation modeling
